# Get Married

**Published:** 1856-09

**Authors:** 


### GET MARRIED.

YOUNG ladies! you will never be satisfied until you do. It is the surest road to a long life and a happy one. There is a thorn in the path now and then, but there is a rose always hard by. Did you never know it before? We will tell you something. We never heard it, nor read it. We found it out. Doctors, you know, are very inquisitive folks, always prying and peeping about, through their own eyes, and other peoples, and when these are not sufficient, they use the microscope, a very favorite instrument with some of them, inasmuch as it enable them

"To see what is not to be seen"

by anybody, except themselves; and full often, they are like the sailor on the look-out: he could not see land exactly, but he could pretty near do it. Well, all at once, one day, this bright idea (so we call it for the present, it may afterwards arise to a fact, for there is a shade of difference between the twain) broke in upon us effulgently. The roses and the thorns of married life are not one and indivisible; they grow on separate stocks, and all that is required to part them, is a good head and a kind heart. There is one difficulty in the way, the thorns are indestructible, but you have only to throw them aside, and if anybody else chooses to pick them up, that is their look-out: every one must see for himself. A bunch of this sort happened to fall to our lot once upon a time, but we can easily account for it, and that is highly satisfactory: we always had weak eyes, and the vicinage thereof is much of a sameness, in a certain phase of the moon. But we fully calculate on repeating the operation; and we intend to have a pair of specs, next time, such as will diminish the blinding glare which Curls and Cotton, in certain conjunctions, attitudes and combinations, do most devastatingly throw around them.

Not long since, a man was head over heels in debt, and he declared that his last speculation left him head over heeler. So, one who tries by marriage to get out of trouble, sometimes gets into greater; but in the large main, marriage is the balm of life, it is the natural condition of human kind, hence, Divinity has ordained it.

The idea which we wished to convey, in connection with the heading of this article, is that while more women than men, in the country at large, die of consumption, yet five hundred married men will die of consumption, while three hundred married women die of it. Therefore, as to women, *marriage, after twenty-five, is a preventive of consumption.*

